# An Atypical Case of Polycystic Ovarian Syndrome and Possible Newly Diagnosed Systemic Lupus Erythematosus Complicated With a Cerebrovascular Accident in a Young Female: A Case Report

**DOI:** 10.7759/cureus.54163

**Published:** 2024-02-14

**Authors:** Divine Besong Arrey Agbor, Derek Ugwendum, Barath Prashanth Sivasubramanian, Maureen A Ojwaka, Sabastain F Forsah, Shakirat Gold-Olufadi, Tochukwu Nzeako, Rita Offor, Nkafu Bechem Ndemazie, Jay Nfonoyim

**Affiliations:** 1 Internal Medicine, Richmond University Medical Center, Staten Island, USA; 2 Infectious Diseases, The University of Texas Health Science Center at San Antonio, San Antonio, USA; 3 Internal Medicine, Employees State Insurance Corporation Medical College & Employees State Insurance Post Graduate Institute of Medical Sciences and Research, Chennai, IND; 4 General Medicine, Universidad de Montemorelos, Nuevo Laredo, MEX; 5 Internal Medicine, Brookdale University Hospital and Medical Center, Brooklyn, USA; 6 Internal Medicine, Christiana Care Hospital, Newark, USA; 7 Internal Medicine, State University of New York Downstate Medical Center, Brooklyn, USA; 8 Pulmonary and Critical Care, Richmond University Medical Center, Staten Island, USA

**Keywords:** auto antibodies, pulmonary tumor embolism, systemic lupus erythematosus, cerebrovascular accident, polycystic ovarian syndrome

## Abstract

Polycystic ovary syndrome (PCOS) is the leading cause of endocrine disorders among females of reproductive age and is linked with autoimmune disorders. PCOS has been associated with autoantibodies such as antinuclear antibody (ANA), anti-thyroid, and anti-Smith (anti-SM). Young patients with PCOS and systemic lupus erythematosus (SLE) have up to a 10-fold increase in stroke. We present a case of a patient with a history of PCOS (on metformin), hypothyroidism, and pulmonary embolism who presented to the emergency room with acute left-sided weakness. She was extensively investigated for risk factors and was eventually diagnosed with a cerebrovascular accident secondary to possible SLE with positive ANA (1:160, nuclear homogenous pattern). The diagnosis of PCOS, coupled with autoantibodies and recurring episodes of thromboembolic events, rendered her case management complex. She received tenecteplase and had thrombectomy done twice because of recurrent thrombotic events during her hospital stay. She passed away on the fifth day post-thrombectomy from a possible massive pulmonary embolism with hemodynamic compromise. There is a need for more research to comprehend the underlying mechanisms of SLE and PCOS to guide the proper management of patients in this situation.

## Introduction

Polycystic ovarian syndrome (PCOS) is the most common endocrine disorder in females characterized by hyperandrogenism, polycystic ovaries, and anovulatory cycles. The World Health Organization (WHO) estimates that about 8-13% of premenopausal women suffer from this condition globally [1]. Nearly all causes of PCOS are due to functional ovarian hyperandrogenism (FOH). Two-thirds of PCOS presentations have typical functional ovarian hyperandrogenism, characterized by dysregulation of androgen secretion with an over-response of 17-hydroxyprogesterone (17-OHP) to gonadotropin stimulation. The remaining PCOS cases with atypical FOH lack an overresponse of 17-OHP, but testosterone elevation can be detected after suppressing adrenal androgen production. About 3% of PCOS patients have a related isolated functional adrenal hyperandrogenism. The remainder of PCOS cases are mild. PCOS has been linked with autoimmune disorders, such as systemic lupus erythematosus (SLE), thyroid disease, hyperprolactinemia, and non-classical congenital adrenal hyperplasia [2,3] and up to 8.6% of females with PCOS have elevated antinuclear antibody (ANA) titers [4,5]. The exact mechanism between PCOS and elevation in ANA is unclear; however, it has been hypothesized that PCOS could raise ANAs through immune hyperactivation and incremental inflammation [3,6]. SLE patients may experience a stroke especially when associated with antiphospholipid syndrome, but repeated thromboembolic events in patients within a short period are uncommon in the literature. Here, we report an atypical case of a middle-aged female presenting with PCOS and possible SLE, who experienced repeated cerebral thromboembolic events. Given that there is scanty data on the occurrence of PCOS and SLE complicating cerebrovascular accidents, our goal is to bring to the fore the severity of this condition and the need for intensive management due to its potential mortality among young adults. 

## Case presentation

A 31-year-old female with a past medical history of PCOS diagnosed (in a different health facility) at age 16, pulmonary embolism eight years ago (received six months of apixaban), obesity, menstrual irregularities, hirsutism, and hypothyroidism presented to the Emergency Room (ER) for sudden onset of aphasia, dysarthria, and left-sided weakness within 70 minutes of symptoms onset. She was on oral contraceptive pills (OCP) for the management of PCOS for 10 years until the episode of pulmonary embolism after which metformin was subsequently commenced. The patient was a nonsmoker and not an alcoholic with no history of recreational drug use. Her family history was non-contributory, and she received her last COVID vaccination two years prior. Vital signs at presentation were 115/75 mm Hg, heart rate of 83/min, and oxygen saturation of 98% on 2 liters/minute through a nasal cannula. On clinical assessment, she was alert and oriented to time, self, and place. She had left-sided facial drooping and hair growing on the chin. Her National Institutes of Health (NIH) Stroke Scale score was 11. Her motor strength on the left upper extremity/limb was about 1-2 on 5. All other muscle groups retained tone and strength, and sensation to pain and touch was preserved. Her laboratory results were significant for hemoglobin of 9.7 g/dl, positive for ANA with titer 1:160 showing a homogeneous pattern. The coagulation and auto-antibody profile are shown in Table 1.

**Table 1:** Coagulation and autoantibodies' profile ANA: Antinuclear antibody

Test	Result	Normal range
ANA	Positive	
ANA titer	1:160	1:40
ANA pattern	Nuclear homogeneous	
Immunoglobin A (IgA)	140	47-300 mg/dl
Immunoglobin M (IgM)	124	50-300mg/dl
Cryoglobulin	Not detected	
C-Antineutrophil Cytoplasmic autoantibody (C-ANCA)	<1.0	<1.0
P- Antineutrophil Cytoplasmic autoantibody (P-ANCA)	<1.0	<1.0
Prothrombin Gene Mutation	Not detected	
Homocysteine	7.8	<10.4 umol/L
Protein C	Not detected	
Protein S	68	60-140
Prothrombin time (PT)	13.9	12-14.8
Partial thromboplastin time (PTT)	29	22.8-36.5
International normalized ratio (INR)	1.05	0.9-1.12
Beta 2-glycoprotein Immunoglobin G	<2.0	<20
Beta 2-glycoprotein IgA	<2.0	<20
Beta 2-glycoprotein IgM	<2.0	<20
Anticardiolipin IgG antibody	<2.0	<20
Anticardiolipin IgM antibody	<2.0	<20
Lupus anticoagulant	Not detected	
Factor V Leiden	Not detected	
Plasminogen inhibitor	68	4-43

**Figure 1:**
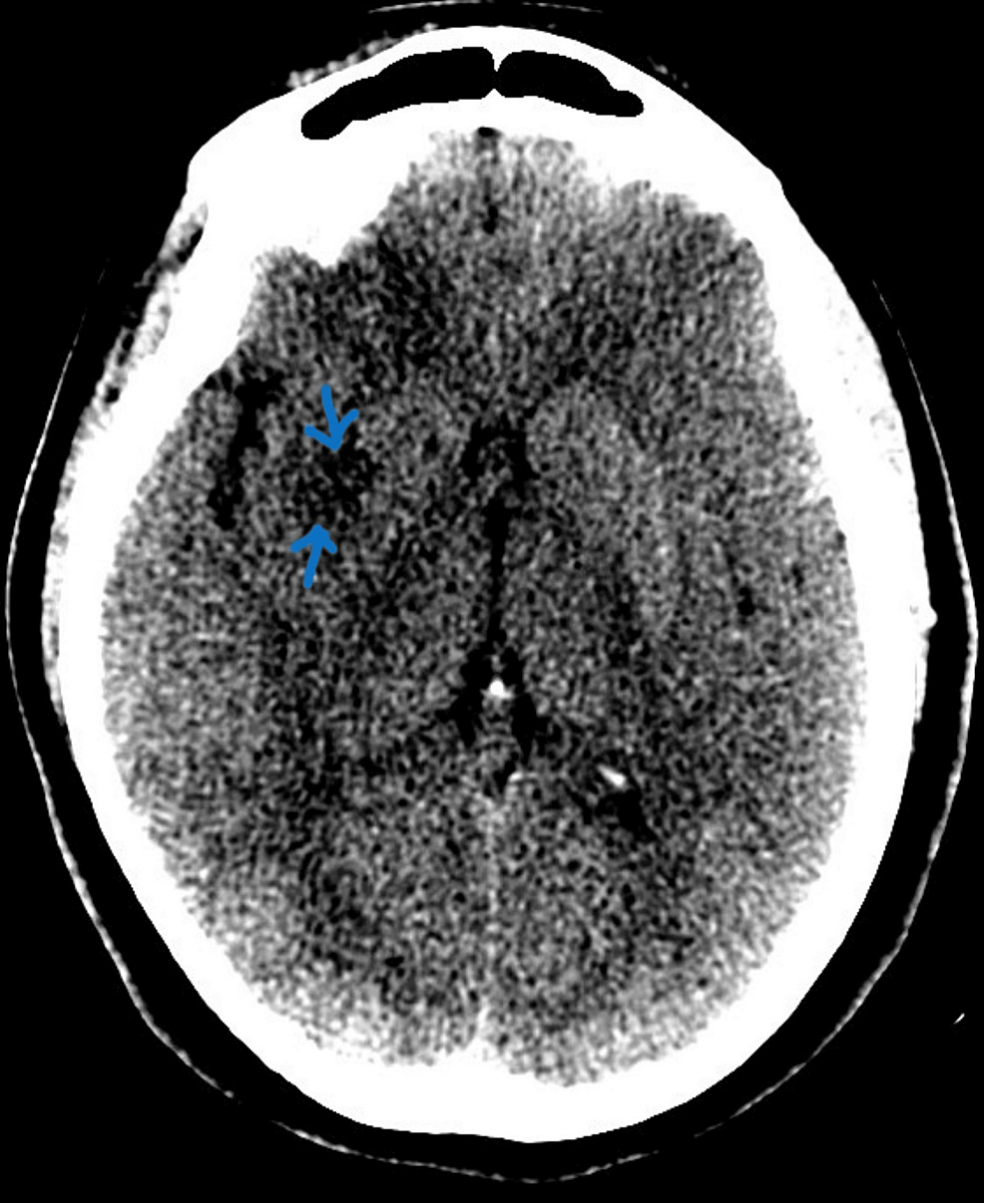
CT scan of the head showing new acute ischemic changes along the right frontal lobe, right corona radiata, and right basal ganglia

**Figure 2:**
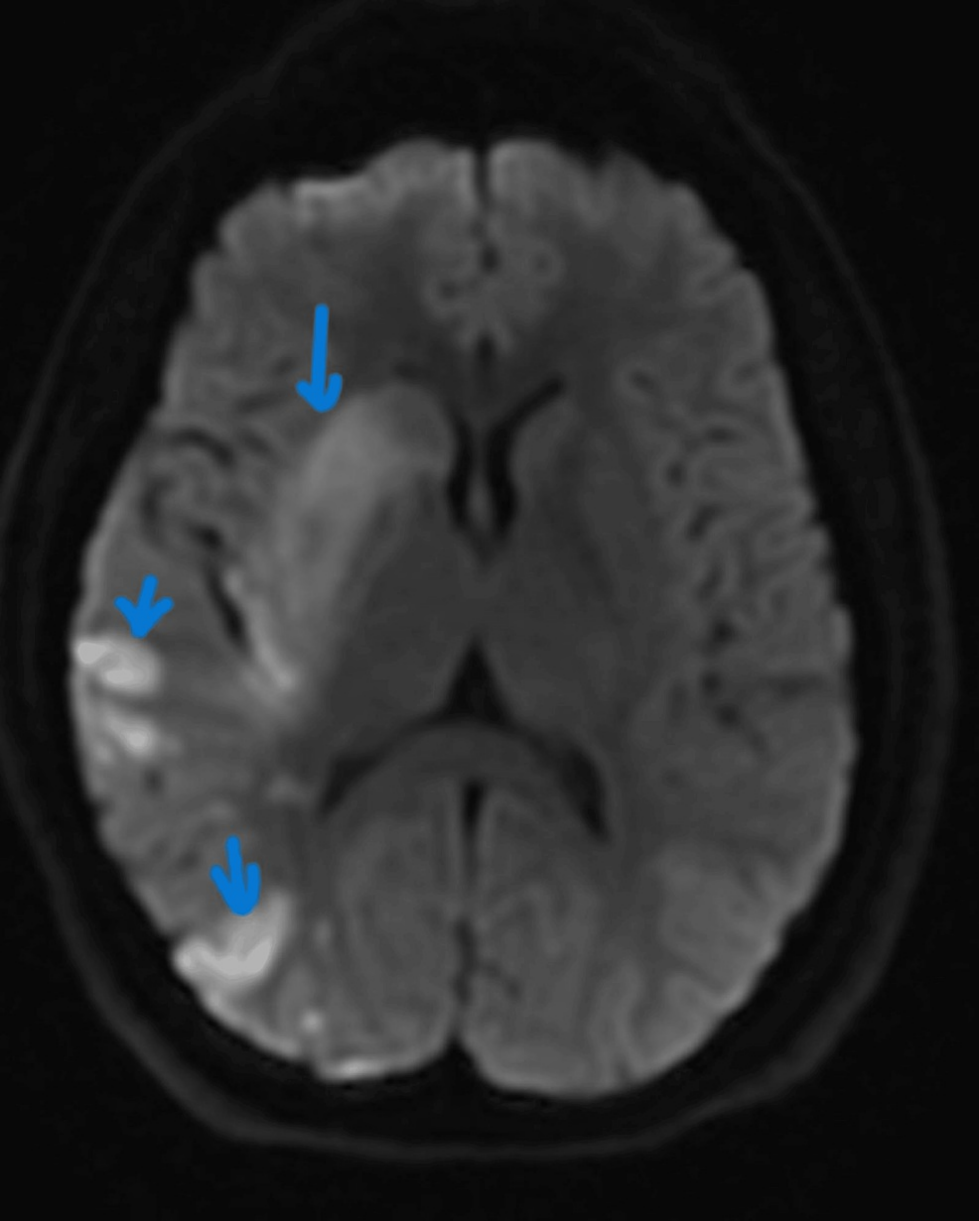
MRI head revealing acute infarcts involving the cortex and white matter within the right frontal, parietal, temporal, and occipital regions with involvement of the right basal ganglia

The transthoracic echocardiogram revealed a left ventricular ejection fraction (LVEF) of 70% while the transesophageal echocardiogram was done to rule out patent foramen ovale. It revealed no intracardiac masses, no focal left ventricular wall abnormalities, no patent foramen ovale, normal diastolic filling pattern, and no major plaques. Computed tomography (CT) head was noted with new acute ischemic changes along the right frontal lobe, right corona radiata, and right basal ganglia and head magnetic resonance imaging (MRI) showed acute infarcts involving the cortex and white matter within the right frontal, parietal, temporal, and occipital regions with involvement of the right basal ganglia. There was a loss of normal flow void along the right M1 segment. Full imaging results are shown in Figures 1, 2 and Table 2. She was diagnosed with an ischemic cerebrovascular accident. She underwent guideline-directed treatment with tenecteplase and 2 x thrombectomies in the middle cerebral artery. The patient was started on clopidogrel and aspirin for secondary prevention of thromboembolic events. Her post-intervention days were marked with initial improvement in her clinical condition. She died on the fifth day post-intervention after going into cardiac arrest.

**Table 2:** Computed tomography (CT), computed tomography angiography (CTA), and magnetic resonance imaging (MRI) findings during the hospital stay

Imaging	Admission Day	Results
CT head with Contrast	Day 1	No acute findings
CTA Head/neck	Day 1	No acute findings
Cerebral angiogram	Day 1	Right middle cerebral artery (MCA) thrombectomy
CT head after thrombectomy	Day 1	Hyperdensity of right frontal cortex
CT head without contrast	Day 2	No intracranial hemorrhage
Repeat CT head without contrast	Day 2	New acute ischemic changes along the right frontal lobe, right corona radiata, and right basal ganglia
CTA head/neck	Day 2	Occlusion along the proximal right carotid terminus and proximal right M1 segment extending into the mid to distal portion. Focal occlusion along the mid to distal right M2 segment
MRI head without contrast	Day 2	Acute infarcts involving the cortex and white matter within the right frontal, parietal, temporal, and occipital regions with involvement of the right basal ganglia. Loss of normal flow void along the right M1 segment
Second cerebral angiogram	Day 2	Mechanical thrombectomy of right middle and anterior occlusion using aspiration and stent retrieval
CT head without head post angiogram	Day 2	Hazy hyperdensities within the right basal ganglia along the Sylvian fissure and the region of infarct likely represent contrast staining
CT head without contrast	Day 3	There was a significant improvement in the contrast staining when compared to the prior scan with residual contrast staining, with no evidence of acute hemorrhage.
MRI head without contrast	Day 4	Acute/ subacute evolving infarct, in the right cerebral hemisphere and cytotoxic edema with new effacement of frontal horn of the lateral ventricle.

## Discussion

PCOS is a heterogeneous disorder characterized by ovulatory dysfunction, hyperandrogenism, and polycystic ovaries [7]. It is characterized by an increased inflammatory state with the secretion of interleukins, chemokines, and adipokines [8] and has been linked with elevated ANA titers [2-5]. However, the exact mechanism behind the elevated ANA titers is unclear but has been linked to immune system activation and increased inflammation [3,6]. In one case report, the patient had PCOS and extensive venous and arterial thrombosis associated with patent foramen ovale [9]. 

About 3-20% of patients with systemic lupus erythematosus develop an acute ischemic stroke within the first five years of diagnosis [10]. SLE induces a hypercoagulable state through various mechanisms involving antiphospholipid antibodies, marantic endocarditis, inflammation-induced atherosclerosis or thrombosis, and cerebral vasculitis [10,11]. SLE has been previously reported to occur with features of an acute cerebrovascular accident as the primary complaint [11-13]. Our patient presented similarly and had a normal echocardiography finding. Central nervous system imaging did not reveal any atherosclerosis or vasculitis. Brain biopsy is considered a gold standard for diagnosing cerebral vasculitis, but this was not done in our case. Current management of patients with SLE and recurrent embolic episodes includes antiplatelet, antiepileptic, and hydroxychloroquine [14]. However, the management of young females with PCOS, SLE, and multiple thromboembolic episodes is not well explained, particularly after thrombolysis and thrombectomy. 

Our patient with background PCOS was diagnosed with a cerebrovascular accident with a new onset of SLE and was treated with thrombolytics, dual antiplatelet therapy, and thrombectomy. Her hospital stay was marked by an initial improvement but suffered a cardiac arrest with hemodynamic instability most likely from a PE and expired after resuscitative measures were futile. Since this is an uncommon presentation, research data are limited in the diagnosis and management of such cases and this report was to highlight this clinical presentation. 

## Conclusions

PCOS is an endocrine disorder with components of auto-immune derangement in reproductive-age women. Our case was that of a young female with PCOS and possible SLE who had an atypical presentation of multiple thromboembolic events during a short period. In such a patient, we lack research data to guide us in prompt diagnosis and management of such cases. Underscoring the importance of early detection and targeted treatment can influence the course of the disease. More research is needed to better comprehend the association of PCOS with SLE, the molecular mechanisms involved in PCOS associated with SLE, and the course of management in these patients. 

## References

[1] Nusrat O (2013). Polycystic ovary syndrome. Free Radic Biol Med.

[2] Escobar-Morreale HF (2018). Polycystic ovary syndrome: definition, aetiology, diagnosis and treatment. Nat Rev Endocrinol.

[3] Azziz R (2018). Polycystic ovary syndrome. Obstet Gynecol.

[4] Mobeen H, Afzal N, Kashif M (2016). Polycystic ovary syndrome may be an autoimmune disorder. Scientifica (Cairo).

[5] Hefler-Frischmuth K, Walch K, Huebl W, Baumuehlner K, Tempfer C, Hefler L (2010). Serologic markers of autoimmunity in women with polycystic ovary syndrome. Fertil Steril.

[6] Pan ML, Chen LR, Tsao HM, Chen KH (2020). Prepregnancy endocrine, autoimmune disorders and the risks of gestational hypertension-preeclampsia in primiparas: a nationwide population-based study in Taiwan. Int J Environ Res Public Health.

[7] Homburg R, Weissglas L, Goldman J (1988). Improved treatment for anovulation in polycystic ovarian disease utilizing the effect of progesterone on the inappropriate gonadotrophin release and clomiphene response. Hum Reprod.

[8] Glintborg D, Andersen M (2010). An update on the pathogenesis, inflammation, and metabolism in hirsutism and polycystic ovary syndrome. Gynecol Endocrinol.

[9] Al Busaidi SA, Al-Farsi M, Al-Maqbali JS, Kashoob MS, Farhan H, Al Rawahi B, Al Alawi AM (2023). Extensive arterial and venous thrombosis in a female with a known untreated polycystic ovarian syndrome: a case report. Cureus.

[10] Shaban A, Leira EC (2019). Neurological complications in patients with systemic lupus erythematosus. Curr Neurol Neurosci Rep.

[11] Habes YM, Ahmaro M, Dwikat Y, Jobran AW, Abunejma FM, Abdulrazzak M (2024). Stroke as an unusual initial presentation of 'malignant' middle cerebral artery infarction involvement in systemic lupus erythematosus. Ann Med Surg (Lond).

[12] Guraieb-Chahín P, Cantú-Brito C, Soto-Mota A (2020). Stroke in systemic lupus erythematosus: epidemiology, mechanism, and long-term outcome. Lupus.

[13] Yazidi MA, Merzouk FZ, Rabii H, Benyoussef H, Bensahi I, Habbal R (2023). Ischemic stroke revealing Libman-Sacks endocarditis: a case report. J Saudi Heart Assoc.

[14] Aryal A, Aryal A, Kshetri D, Karki S, Khadka S (2023). Recurrent left ischemic stroke in a patient with systemic lupus erythematosus: a case report. Clin Case Rep.

